# How to modernize without compromising history: a case study of the Franzello Aeromedical Library's journey in updating collections, capabilities, and facilities

**DOI:** 10.5195/jmla.2024.1792

**Published:** 2024-04-01

**Authors:** Melanie Lazarus, Theresa Bedford, Sara Craycraft, Elizabeth Irvine, Cathy Stahl, Kristen Young

**Affiliations:** 1 melanie.lazarus@us.af.mil, Dean and Professor of Medical Education, United States School of Aerospace Medicine, Air Force Research Laboratory, Wright-Patterson Air Force Base, OH; 2 Assistant Dean of Operations Research & Evidence Based Medicine United States School of Aerospace Medicine, Wright-Patterson Air Force Base, OH; 3 Librarian, United States School of Aerospace Medicine, Franzello Aeromedical Library, United States School of Aerospace Medicine, Wright-Patterson Air Force Base, OH; 4 Librarian, United States School of Aerospace Medicine, Franzello Aeromedical Library, United States School of Aerospace Medicine, Wright-Patterson Air Force Base, OH; 5 Library Technician, United States School of Aerospace Medicine Franzello Aeromedical Library, United States School of Aerospace Medicine, Wright-Patterson Air Force Base, OH; 6 Branch Chief, Franzello Aeromedical Library, United States School of Aerospace Medicine, Wright-Patterson Air Force Base, OH

**Keywords:** Library modernization, library redesign, library capability, collection weeding, aerospace medicine

## Abstract

**Background:**

Academic libraries play a significant role in the student learning process. However, student needs and preferences as well as new paradigms of learning are driving libraries to transition from quiet book repositories to places of collaboration and open information. This descriptive, mixed methods case presentation explores the transition of one library, the United States Air Force School of Aerospace Medicine Franzello Aeromedical Library, in three key areas: collection, capability, and facility. Due to the niche subject matter and audience the library serves, this case also describes how the Franzello Aeromedical Library's distinct collection and capability remained intact throughout modernization.

**Case Presentation:**

The Franzello Aeromedical Library's modernization project aimed to augment the library as a cutting-edge resource supporting USAFSAM's education, consultation, and research mission to equip Aerospace Medicine Airmen with the skills and knowledge for healthcare delivery in austere environments. This project was approached using five phases: 1) best practices baseline, 2) baseline evaluation of library visitor needs, 3) collection weeding, 4) capability, and 5) space design and construction.

**Conclusion:**

As a result of this complex two-year project, several recommendations were gleaned. Use the effort as an opportunity to market library services to new audiences. Ensure all stakeholders are at the table from day one and in perpetuity to save time, and consider using purposeful decision-making models, such as Courses of Action, to make tough calls. Be prepared for delays by padding your timeline and compromise where necessary to keep the project alive. Finally, the authors recommend using in-project discovery and findings to plan for future need justification.

## BACKGROUND

Libraries are unique and play a variety of roles for their users [1]. Special libraries face challenges in adapting to the ever-changing needs of their users and evolving technologies. Unlike general academic libraries, special libraries house specific collections tailored to niche subject areas. These focused collections can become outdated, cluttered, and underutilized without periodic evaluation and modernization. The twenty-first-century library must continually reinvent itself as an environment that cultivates curiosity, participation, teamwork, and continuous education [2]. While renovation and modernization both involve improving the physical space, infrastructure, and user experience, modernization efforts go beyond basic renovations to holistically transform library services, collections, and workflows along with evolving user needs and best practices.

In a special report funded by National Library of Medicine, National Institutes of Health, and Department of Health and Human Services, Lynn, FitzSimmons, and Robinson wrote that library users will have less time, more needs, and a desire for information rapidly [3]. Thibodeu also suggested that future roles of librarians should be expanded to include partner, collaborator, advisor, evidence-based medicine expert, educator, and information filter. Librarians should be informers of system designs and make libraries more valueable [3].

Currently, the National Library of Medicine is underway with a six-year renovation effort that includes converting underutilized space into a variety of team huddle spaces, state-of-the-art training areas, a central reading room, workstations, and offices [4]. Its modern design is expected to improve the workplace experience and focus on team collaboration [4].

Libraries are service organizations that embody their institution's mission and vision [5]. Librarians must be stewards of the library building and remain proactive and creative in negotiating space for the library and its services [6]. Library leaders must recognize the need to build and sustain libraries through activities like collection assessments, user surveys, digital archiving, advocacy, and strategic planning [7]. To meet this need, the United States Air Force School of Aerospace Medicine conducted a descriptive, mixed methods case presentation that explored the modernization of the Franzello Aeromedical Library (FAL) in three key areas: collection, capability, and facility. Due to the niche subject matter and audience the library serves, this case also describes how the FAL's distinct collection and capability remained intact throughout modernization.

## CASE PRESENTATION

The Franzello Aeromedical Library (FAL) is the most extensive aeromedical library in the world, with over 300,000 volumes [8]. Originally known as the AeroMedical Library, the Franzello Aeromedical Library was rapidly moved in its entirety from Brooks City-Base, Texas to Wright-Patterson Air Force Base near Dayton, Ohio after the relocation of its associated academic institution, the United States Air Force School of Aerospace Medicine (USAFSAM) under the 711th Human Performance Wing (711HPW). On June 24, 2011, the library was renamed the Franzello Aeromedical Library (FAL) in honor of its former director, Joseph Franzello. During the dedication, USAFSAM's commander, Colonel Christian Benjamin, noted the continued mission of the FAL as “not to lose the aviation medicine lessons that we learned over the years” [9].

Due to time restrictions, the FAL's holdings were moved in their entirety, resulting in a collection that consumed most of the library's 13,000 square feet from day one despite being in a newly constructed building. By 2017, the FAL completely outgrew the existing space, and lack of weeding resulted in an abundance of items no longer needed in the collection and a space not suitable for students or faculty. Shelving reached the entryway blocking easy entry and most natural light. There was no space for a formal circulation desk, and student study spaces were crammed between the stacks.

Discussions on library modernization began in 2015, but the project had neither a champion nor funding. It was not until the fall of 2020, after the hiring of USAFSAM's first civilian dean, Dr. Melanie Lazarus, and the development of the school's first strategic plan, that the library team was officially tasked and funded by the Office of the Dean to complete modernization of the FAL.

The FAL's modernization project aimed to augment the library as a cutting-edge resource supporting USAFSAM's education, consultation, and research mission to equip Aerospace Medicine Airmen with the skills and knowledge for healthcare delivery in austere environments [10]. Under the leadership of its library director, Ms. Kristen Young, this project was approached using five phases: 1) best practices baseline, 2) baseline evaluation of library visitor needs, 3) collection weeding, 4) capability, and 5) space design and construction.

## PHASE 1: BEST PRACTICES BASELINE

In 2017, members of the USAFSAM Office of the Dean visited six libraries including academic, federal, public, and research institutions to benchmark modernization best practices (Table 1). These institutions were chosen because they: 1) represented libraries with a similar population and comparable resources, 2) had completed a recent large-scale modernization, or 3) were located within the local area.

**Table 1:** Library Best-Practice Site Visit Results

Library	Location	Findings
Herman B. Wells Library, University of Bloomington	Bloomington, Indiana	The University of Bloomington's newly renovated library created separate glass enclosed areas specifically for faculty collaboration to discuss curriculum enhancements without student ears and a quadrant matrix to ensure that library traffic matched physical space needs.
McDermott Library, United States Air Force Academy (USAFA)	Colorado Springs, Colorado	The United States Air Force Academy's modernization effort conducted a user-survey on modernization and found that faculty, staff, and cadets desired dedicated work, study, and research space; extended hours with 24–7 access; and enclosed collaborative rooms. The modernization effort culminated in the library's ability to function as a “marketplace” for the exchange of knowledge and ideas, and a repository of accumulated knowledge. The library reorganized 130k sq ft. including a physical space for a 150k volume active collection with additional closed stacks and an archive collection, seating for 1,200 patrons with spaces for both focused work and informal interaction, reading rooms, multi-use meeting spaces, an expanded academic success center, experimentation spaces to support digital scholarship, and enhanced access and visibility to special collections.
Air Force Medical Service (AFMS) Virtual Library	Virtual	The virtual library had resources to support the clinical needs of hospitals. It has point of care tools and multiple databases.
D'Azzo Research Library, Air Force Research Laboratory (AFRL) and the Air Force Institute of Technology (AFIT)	Dayton, Ohio	The AFRL library encompasses 40k sq ft and was awaiting funding to complete a re-design of its physical space based on a space utilization survey to include additional conference space and a large collaboration area.
Wright Brothers Institute Innovation and Collaboration Center/Tec^ Edge the Air Force Research Laboratory's (AFRL) Discovery Lab	Dayton, Ohio	The Wright Brothers Institute was designed to connect people, support collaboration, and hold meetings. It has an idea lab and a command center for staff with rooms that seat 20–50 persons depending on configuration. The center of the building is home to a café with beverages and snacks available for purchase, three microwaves, a toaster, toaster oven, and a refrigerator for guest use.
Air Force Libraries (AF)	Nationwide	Seventeen base libraries across the Air Force underwent modernization projects, highlighting Department of Defense support and justification for a fresh physical space to connect with users with televisions, media screens, furniture, etc.

Findings were presented to the FAL team to inform the modernization effort in collection, capability, and facility. The team determined that the library needed to be functional to meet today's mission. Best practices included the need to update the collection through digitization and updated materials and to re-design the physical space to support the needs of current students and staff for comfort, relaxation, and collaboration.

## PHASE 2: BASELINE EVALUATION OF LIBRARY VISITOR NEEDS

In order to identify areas within the library that needed to be updated, the FAL released an anonymous, online modernization survey to students and faculty and established a team to guide the modernization project.

### FAL Modernization Survey

The FAL Modernization Survey was developed by librarian staff and administered using LibWizard software. All surveys were voluntarily completed, and participants could skip questions if desired. Participants were asked to rate the importance of different aspects of the library space, technology, and librarian services (see Table 2). Responses were scored on a scale from 1 (not important) to 5 (extremely important), and mean responses for each item were calculated. Questions in predetermined checklist and open text box formats were also included to collect participant preferences such as library hours, librarian assistance, and physical spaces.

**Table 2:** Franzello Aeromedical Library Modernization Survey

Survey Item n=30	Mean Response
Please rate the importance of patron computers to you	3
Please rate the importance of technology to you	3.75
Please rate the importance of study space to you	4.25
Please rate the importance of meeting space to you	2.5
Please rate the importance of the general collection to you	3.83
Please rate the importance of the journal collection to you	4.67
Please rate the importance of databases to you	4.54
Please rate the importance of E-books (not course books)	3.62
Please rate the importance of librarian availability	5
Please rate the importance of librarian capabilities to you	5
Please rate your satisfaction with library hours	4.58

A total of 30 surveys between December 2020 and November 2022 from researchers (n=19), consultants (n=2), nurses (n=1), contractors (n=2), staff members (n=4), and non-affiliated users (n=1) were completed. Overall, responses suggested that library patrons to the FAL most valued librarian availability and capability (M=5), the journal collection (M=4.67), databases (M=4.54), and personal study space (M=4.25) highly. Meeting space (M=2.5) was rated lower.

Anecdotal library patron comments indicated that patrons desired library space upgrades to include secluded areas for private studying; smartboards; designated areas and equipment for video conferencing; hands-on technology such as virtual reality headsets, gamification tools, and makerspaces.

### Modernization Team Meetings

To guide the modernization project, the FAL built a modernization team of 12 subject matter experts and stakeholders from across the 711HPW. A project charter was developed to establish the scope of work, requirements, deliverables, and project objectives. More specifically, the charter defined the mission requirements to preserve library archive materials and update the library space. Specific goals of the renovation such as assessing holdings and current space, enhancing the library's layout, and preserving archive media were provided. The final document was approved by the dean and deputy commander to provide guidance and hold the team accountable.

The team met twice a month for one hour from February 2019 through December 2022 to assess: 1) the standing of current and outdated physical holdings, 2) physical space needs, 3) the shift to digitization, and 4) renovation needs while adhering to Department of the Air Force building constraints.

Although the response rate for surveys was extremely low due to self-selection and the ability to skip questions in the survey, this data provided the FAL team with a starting point for stakeholder discussion. Patron comments, best practices seen at other libraries, and resource availability was also used. Ultimately, the team concluded that the modernized space must include comfortable study and research areas, a digitized collection, both private and group collaboration rooms, rest/relaxation areas, reconfigurable presentation monitors, a Mezzanine/2nd floor, and glass walls to sustain daylighting in the space.

## PHASE 3: WEEDING THE COLLECTION

A review of the physical collection was conducted to reduce the footprint of the stacks. The team opted to keep, discard, or donate items using the following guidelines: 1) holdings available in other libraries, 2) usage statistics, 3) duplicate copies, 4) outdated materials, 5) electronic availability, and 6) not considered to be pertinent to aerospace medicine.

Circulating books and journals were targeted for weeding while the archives, office collections, and textbooks remained unaltered. The FAL elected to keep a large portion of journal holdings to continue supporting the robust interlibrary loan program. Moreover, to maintain a more accurate representation of actual holdings, the FAL completed an extensive reclamation project with the Online Computer Library Center and reviewed both the physical holdings and catalog records in WorldCat/DOCLINE. These were time-stamped, and holdings no longer remaining in the collection were deleted.

The circulating book collection was ultimately reduced by 60% to 13,380 items. Although journal deselections were tracked by title not by the number of volume holdings, approximately 30% of the journal collection was reduced to a total of 1,318 titles. Items were withdrawn and donated to other libraries and the remaining collection was shifted to its new location.

## PHASE 4: CAPABILITY

Next, the FAL team focused on capabilities, both in services and personnel. The library did not have a digital presence necessary to package and showcase content and resources, but the library lacked funding to purchase new systems.

As a creative solution, the FAL team applied for an Air Force-based research grant to provide both the financing and evidence-based approach needed to select the most appropriate platforms for librarians and patrons alike. The team purchased one-year licenses for four platforms using the $204K awarded. Fortunately, the timing was incredibly apropos as the technology allowed the FAL to offer off-campus resources during the COVID-19 pandemic.

As part of the research project, the FAL team moved all electronic content from an intranet website to a popular library content management system (CMS). In addition to an updated look for marketing purposes, this CMS provided new features including ticketing queues for services such as literature reviews and interlibrary loan requests, a help desk, custom information hubs, three customer service dashboards, and usage analytics.

## PHASE 5: SPACE DESIGN & CONSTURCTION

Facility modernization began once the FAL team relocated items to the archive and removed selected items for donation, discard, and disposal.

**Figure 1:**
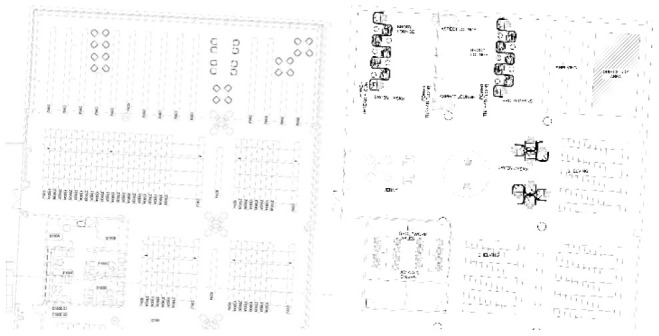
Comparison of Franzello Aeromedical Library Versus Modernized Floor Plan

The original library space was approximately 13,000 square feet with a separate administration area including three offices, two cubicles, and a reference counter (Figure 1). It had a separate technical reports room, archives area, and a reading room with more than 2,000 square feet of compact and fixed shelving. There were three computer kiosks with 12 workstations that provide customers with wired internet access.

**Figure 2:**
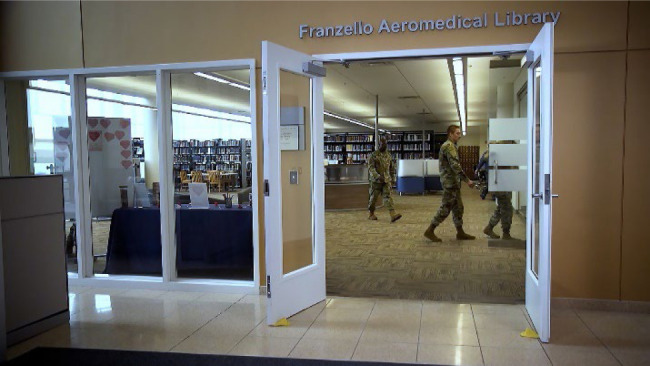
New entrance to the FAL with featured collection and circulation desk. Previously, rolling and fixed shelving blocked visibility into the library from the front entrance glass and prevented sunlight exposure from the North-side windows

Removal of the nine double-sided, free-standing shelving units located under the high ceiling was completed by the in-house USAFSAM's Facilities Division (Figure 2). Removal of the 20 double-sided Spacesaver High-Density Storage Rolling Shelving Units in the FAL's entryway required an external contractor due to being secured into the original concrete flooring. Before relaying the carpet, the contracting team had to return more than once to ensure the flooring was level and not an unnecessary tripping hazard.

**Figure 3:**
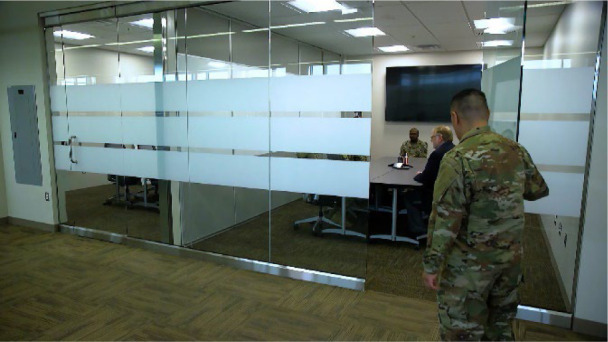
New state-of-the-art collaboration room with seating for 16 patrons. The room can be separated into two spaces using a movable dividing wall and is outfitted with two computer workstations, wall-mounted screens, dry-erase boards, and virtual collaboration technology.

The construction team created a new collaboration space with a glass front and two entry doors for student and faculty engagement (Figure 3). This space included a folding wall divider to split the room into two for user flexibility. It was also outfitted with folding conference tables, dry-erase boards, two 86-inch screens, two video teleconferencing units, and two instructor stations.

Having recently completed their own modernization effort of similar scope for a similar patron audience, USAFSAM turned to the United States Air Force Academy McDermott Library for inspiration, who had recently partnered with an experienced, national architecture firm with healthcare and library redesign experience. The firm had reviewed USAFA's McDermott Library to create a plan that included project guidelines, goals and strategies; summary of findings; building recommendations; concept development; implementation and budget. The previous space reflected late twentieth-century models and did not meet user demands that define a twenty-first-century library with technology-rich, flexible study and learning spaces. Guidelines included: provide a twenty-first-century learning environment; develop a functional, integrated, sustainable, and future-ready library; promote flexibility and interdisciplinary collaboration; protect the architectural heritage and key historical features; reestablish monumental circular stairs as a focal point; leader in energy and environmental stewardship. Cadets desired varied space options such as enclosed collaborative study rooms and places for individual work. To accommodate these changes in the existing 130,000 sqft space, the McDermott Library allotted 25% to 50% of the total square footage for services, while decreasing the space allocated for print collections from 50% to 30% of the total. Similar to FAL, this decision was possible due to 75% of the collections not being circulated in the past 15 years.

The in-person visit to the McDermott Library at the United States Air Force Academy largely inspired the FAL's furniture selection. With 40,000 USAFSAM patrons annually, the modernization team believed that the furniture design should be ergonomically designed, timeless, durable, and versatile. In addition, the modernization team made selections based on inclusivity of body types and variable studying styles. The furniture ultimately selected was a combination of private, pair, and group study spaces with ergonomic considerations and e minimalist look, arranged to create defined spaces. These new spaces included: 1) formal entry with seating; 2) circular style reference desk for two librarians; 3) high-performance workspaces with built-in lighting, power ports, cubbies for personal belongings, footstools, and an ergonomic chair; 4) high back sofa booths with integrated work surfaces, power, and panel-mounted monitors to review work with a colleague; 5) comfortable living room “fireside chat-style” seating area with couches, footstools and chairs on accent rugs; 6) enclosed ergonomic pod desks with power and lighting for studying; and 7) oversized swivel chairs with cupholders and large rolling dry erase boards.

The team also designated a climate-controlled room for archived materials and office space for assigned staff members.

**Figure 4:**
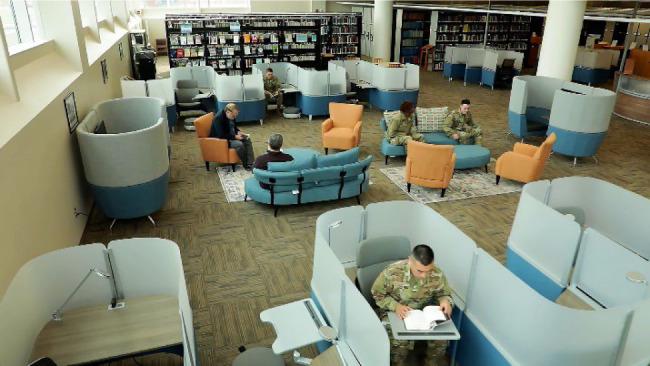
Cleared the North side of the library, previously occupied exclusively by rolling and fixed shelving. Furniture pictured includes individual study lounges with ergonomic chairs, footstools, bag cubbies, swivel lap desks, lamps, and power stations; workstations with fixed desks, chairs, and lamps; digital collaboration spaces for two with desks, mounted monitors, and HDMI connections; and a relaxation space with armchairs, sofas, ottoman, and rug.

While FAL staff was reluctant to begin a project of such great scope, magnitude, and cost, the overall benefit of creating a smaller, more streamlined, and mission-focused physical collection was quickly recognized. FAL now has a much more versatile space with a large, open floor plan that can be easily reconfigured, positioning USAFSAM as more of a learning commons in support of an agile work environment that sparks collaborative learning, scholarship and engagement where visitors can come to connect with one another (Figure 4).

Furthermore, this additional space has given FAL the capacity to host both internal and community-based events. Since the recent completion of the renovation, FAL has hosted library-related instructional sessions, collaboration events, book clubs, open houses, holiday engagements such as a trick-or-treat event for families, large courses, and student breakout sessions. Additionally, FAL has added a small number of items to its collection that support collaboration and stress reduction such as leisure reading titles, games, coloring, and sensory materials, share a card with your colleague, and affirmation cards. The affirmation cards have become very popular and are being utilized in morning meetings and classes to further reflect and connect with one another.

## DISCUSSION

The FAL modernization project took place over two years with dedicated work from a team of subject matter experts and leaders within the 711HPW. The FAL required a complete re-design to encourage learning, collaboration, socialization, and quiet working. Consistent with previous literature and survey results, a centralized circulation desk, group collaboration rooms with large glass windows and multi-media tools, comfortable individual carousels, and a seating area for socialization were included [4,8]. As a result of this long and complex project, the authors have several major recommendations for those seeking to undertake a similar effort.

### Capitalize on the Redesign for Marketing

To market the new space to patrons, the modernization project culminated in a grand re-opening held on Tuesday, March 21, 2023. Sixty-five attendees were provided an overview of the modernization effort, invited to view historical items from the archive, and offered a tour of the space. Attendees spoke highly of the reconfigured space and décor specifically.

Furthermore, the FAL team established a marketing plan that will be reevaluated every six months to promote its services, resources, and space in perpetuity.

### Facilitate Multi-Level Stakeholder Engagement

A project of this magnitude requires robust teamwork and frequent meetings. Modernization team composition must be considered upfront, and all stakeholders with a vested interest in the project need to be invited to the table on day one. The FAL team found that a strong champion in a position of authority, such as the dean, was needed to advocate for funding and sustain momentum for the multi-year effort. It is also highly recommended that IT and facilities be regular modernization meeting participants to avoid timeline delays and ensure feasibility discussions occur. For continuity, meetings should include an agenda specifying old and new agenda items. Touchpoints on complex tasks should be discussed frequently to prevent overlooked details.

### Use Purposeful Decision-Making Methodology

A purposeful methodology is recommended to ensure a holistic approach to decision-making.

The authors recommend using Courses of Action (COAs) and Plan of Action & Milestones (POAMs), unique high-level stakeholder engagement formats widely used throughout the Department of Defense. These informal tools convey a set of defined options and tasks to enable leadership to make an informed decision rapidly that will ultimately lead to desired outcomes [11]. COAs are written documents used to highlight executable options including a proposed action, timeline, resources, manpower, and costs to influence a predicted outcome, while POAMs are figures that illustrate action steps along a timeline. Following the same format, academic institutions can use COAs in a similar manner by systematically evaluating the pros and cons of each option that can be feasibly carried out to achieve the desired outcomes.

Moreover, the FAL used baseline recommendations from public and military-specific libraries; current student, instructor, researcher, and visitor surveys; and team meetings with a diverse group of subject matter experts (SMEs). Member checking was conducted through team meetings with the dean, known in the Air Force as “vector checks,” to confirm findings and overall modernization direction. The FAL team ensured that a distinct collection remained by further consulting departmental and operational field subject matter experts for unique collection areas. Data triangulation and member checking was used to ensure multiple points of observation and interpretation [12]. This methodology was critical to increase our trust in the decisions made, ultimately enabling the modernization effort to be seen to fruition without losing the heritage, culture, and operational mission of the library.

### Expect Delays and Be Flexible

Building projects are prone to delays. One must remain flexible and patient and pad timelines to facilitate stakeholder return on expectations. Supply chain issues due to COVID-19 and building requirements significantly impacted both project direction and timeline. To maintain momentum, the team selected a COA that would allow them to make significant changes in a timely manner. Although not the ideal COA, it was the most feasible. For awaiting items, as much preparatory work as possible was completed in anticipation of sufficient funds and leadership approval. Additionally, due to the high expense of the project in a resource-constrained environment, financing had to be split amongst several stakeholders including the Office of the Dean, IT, and Facilities. The team also used as many low-cost options as possible such as the use of in-house recycling using Defense Logistics Agency Disposition Services for select textbooks and in-house muscle through a “library clean out day.”

### Uncover Future Needs

Finally, it is recommended to use in-project discovery and findings to plan for future need justification. In the final year of this effort, a particular concern was uncovered during the construction phase: a waterline running throughout the library's archive which could threaten historic materials and one-of-a-kind items. Fortunately, the probability of water damage was determined to be extremely low due to the waterline being a secondary feed activated only in times of an emergency. Thus, leadership opted to wait on this final portion of the space project due to lack of funding. However, this discovery enabled the team to plan for their next project and provided the justification needed to continue to innovate and modernize.

Future modernization projects should consider the findings and lessons learned from the FAL modernization effort to build a team, gather the best data and resources, communicate effectively, and address challenges early.

As a result of this modernization, the FAL library will play a much larger role in the USAFSAM community moving forward. Plans are already underway to add more historic artifacts to the library's collection, sourced from over 100 years of aerospace history in buildings throughout Wright Patterson Air Force Base. FAL library leadership is already engaging with community libraries in a new way, with plans for rotating unique showcase collections monthly. More courses are being hosted in the space regularly, and school engagement activities such as a recent event centered around the solar eclipse are being executed. The next major renovation project under consideration is a complete overhaul of the archives, which would provide a more organized and secure space for one-of-a-kind items.

Since the completion of the modernization effort, the FAL has seen a 50% increase in foot traffic with a gate count of 16,182 in 2022 to 24,276 in 2023. At a time in which many libraries are struggling to even maintain their pre-pandemic in-person throughput, the success of the FAL is an ideal case study for patron engagement success. In summary, the FAL modernization has opened the door to increased productivity, collaboration, and networking within the organization, while maintaining the FAL's distinct collection, historic facility, and capabilities. Despite being a cumbersome and often stressful endeavor requiring tremendous time and resources, revitalizing a library can be worth the effort. Libraries interested in better engaging with existing patrons, attracting new audiences, making their space more relevant, and better showcasing their capabilities should considered a similar effort as a way forward for future success.

## DISCLAIMER STATEMENT

The views and opinions presented herein are those of the authors and do not reflect the official guidance or position of the United States Government, the Department of Defense, the United States Air Force, or the United States Space Force.

## DATA AVAILABILITY STATEMENT

Data associated with this article is property of the United States Air Force School of Aerospace Medicine and cannot be made publicly available. Access to the data can be requested from the corresponding author.

## AUTHOR CONTRIBUTIONS

Melanie M. Lazarus: Conceptualization; methodology; formal analysis; writing—original draft; supervision; project administration. Theresa Bedford: Methodology; formal analysis; writing—original draft. Sara Craycraft: Investigation; Elizabeth Irvine: Investigation. Cathy Stahl: Investigation. Kristen L. Young: Funding acquisition; investigation; supervision; formal analysis; writing—review and editing.
